# The Effect of Bacteria-to-Calcium Ratio on Microbial-Induced Carbonate Precipitation (MICP) under Different Sequences of Calcium-Source Introduction

**DOI:** 10.3390/ma17081881

**Published:** 2024-04-19

**Authors:** Teng Zhao, Hongxiu Du, Ruihua Shang

**Affiliations:** 1 College of Civil Engineering, Taiyuan University of Technology, Taiyuan 030024, China; 13467169066@163.com; 2 College of Architecture, Taiyuan University of Technology, Taiyuan 030024, China; shangruihua@tyut.edu.cn

**Keywords:** MICP, sporosarcina pasteurii, biomineralisation, calcium carbonate, bacteria-to-calcium ratio

## Abstract

To explore the effects of the introduction order of calcium sources and the bacteria-to-calcium ratio on the microbially induced calcium carbonate precipitation (MICP) product CaCO_3_ and to achieve the regulation of CaCO_3_ crystal morphology, the mineralisation products of MICP were compared after combining bacteria and calcium at ratios of 1/9, 2/9, 3/9, 4/9, 5/9, and 6/9. A bacterial solution was combined with a urea solution in two calcium addition modes: calcium-first and calcium-later modes. Finally, under the calcium-first addition method, the output of high-purity vaterite-type CaCO_3_ was achieved at bacteria-to-calcium ratios of 2/9 and 3/9; under the calcium-later addition method, the output of calcite-type CaCO_3_ could be stabilised, and the change in the bacteria-to-calcium ratio did not have much effect on its crystalline shape.

## 1. Introduction

Many organisms in nature have some mineralisation, such as the bones and teeth of mammals, abalone (mollusc) shells, and barnacle (arthropod) shells [1]. The phenomenon of biomineralisation is caused by micro-organisms with mineralising properties. A variety of mineralisation models exist in microorganisms, including nitrate reduction models, sulphate models [2], and denitrification models [3]. The model of carbonate precipitation through urea hydrolysis by urease microorganisms [4] is the most direct and controllable of the MICP processes, capable of generating large quantities of carbonate in a short period of time. Thus, urease microbial hydrolysis of urea is a widely used model of microbial mineralisation in which urease-producing microorganisms, represented by *Sporosarcina pasteurii*, metabolically secrete urease and hydrolyse urea in the environment [5,6,7]. Urea, as an organic small molecule, can enter the bacteria’s cell by free diffusion. When the concentration of urea in the environment is high, it will influx into the bacterial interior in large quantities and catalyse hydrolysis under the action of urease inside the bacterium [8]. At physiological pH, carbonic acid protons are dissociated, and ammonia molecules are protonated by water molecules, which leads to a net increase in pH [9]. The whole reaction process is shown in reaction Equations (1)–(4) [8,9].

(1) $${\mathrm{CO}(\mathrm{NH}}_{2}{)}_{2}+H_{2}O\overset{\mathrm{urease}}{\to }{\mathrm{NH}}_{3}+{\mathrm{NH}}_{2}\mathrm{COOH}$$

(2) $${\mathrm{NH}}_{2}\mathrm{COOH}+H_{2}O\to H_{2}{\mathrm{CO}}_{3}+{\mathrm{NH}}_{3}$$

(3) $$H_{2}{\mathrm{CO}}_{3}\to 2H^{+}+{\mathrm{CO}}_{3}^{2-}$$

(4) $${\mathrm{NH}}_{3}+H_{2}O⇌{\mathrm{NH}}_{4}^{+}+{\mathrm{OH}}^{-}$$

Calcium carbonate precipitation is a fairly simple chemical process controlled by four key factors [10]: calcium concentration, concentration of soluble inorganic carbon, pH, and the presence of nucleation sites. Calcium carbonate precipitation requires sufficient concentrations of calcium and carbonate ions so that the product of the ionic activities (α) of the two ions exceeds the dissolution equilibrium constant (K_SO_) of calcium carbonate, as shown in Equations (5) and (6). By the ratio of the ionic activity product to the dissolution equilibrium constant, the saturation of the system can be defined as Ω; when Ω > 1, the solution system reaches a supersaturated state and may precipitate calcium carbonate. Under natural conditions, the precipitation process described above would be very slow, but microorganisms can control or induce calcium carbonate precipitation by altering any of the key factors mentioned above that affect calcium carbonate precipitation, resulting in the production of large amounts of calcium carbonate in a short period of time.

(5) $${\mathrm{Ca}}^{2+}+{\mathrm{CO}}_{3}^{2-}\to {\mathrm{CaCO}}_{3}$$

(6) $$\Omega =\alpha ({\mathrm{Ca}}^{2+})\alpha ({\mathrm{CO}}_{3}^{2-}{)/K}_{\mathrm{so}}{(K}_{{\mathrm{so}25}^{{}^{\circ}}C}=4.8\times 10^{-9})$$

The ability of *Sporosarcina pasteurii* to induce calcium carbonate precipitation is mainly attributed to two main features. The most important feature is the ability of *Sporosarcina pasteurii* to secrete highly active urease, which catalyses the decomposition of urea within the bacterium by virtue of its highly efficient urease activity [11,12,13,14,15], rapidly increasing the carbonate concentration and pH of the microenvironment around the bacterium; second, it has been shown that the surface of *Sporosarcina pasteurii*, including the cell wall and extracellular polymers, has a greater number of negative surface charges and thus can more strongly adsorb cations than the surface of nonmineralised bacteria [16], so positively charged Ca^2+^ in the environment can be adsorbed in large quantities. Under high concentrations of carbonate and alkaline conditions, Ca^2+^ then settles to form CaCO_3_ crystals using cells as nuclei.

It is now widely believed by many scholars [17,18] that *Sporosarcina pasteurii* mainly plays two roles in the whole mineralisation process: a. decomposing urea from the environment to promote the formation of an alkaline environment and the production of carbonate ions, which provide the necessary chemical conditions for the biomineralisation reaction; b. adsorbing Ca^2+^ from the environment to provide the necessary metal ions for the biomineralisation reaction and to provide nuclei for the crystallisation of mineralised material. However, in 2018, Zhang [19] carried out in situ, real-time observation of the biomineralisation of *Sporosarcina pasteurii* at the single-cell level. During MICP, nanoscale calcium carbonate was first formed on extracellular polymers on the cell membrane and tightly bound to the bacteria’s cell surface, but most of the submicron and even micron-sized mineralisation products continued to be mineralised as nanoscale calcium carbonate spheres released into the culture medium. However, it is undeniable that the bacteria’s cell surface plays an important role in mineralisation, and the presence or absence of bacteria has an important influence on the crystal morphology and crystal structure of the mineralisation products.

Yang [20] investigated the role of heterotrophic nitrifying bacteria in the MICP process, providing a theoretical basis for the application of microorganisms to achieve simultaneous nutrient removal and phosphorus recovery from wastewater. Recent studies [21] have shown the degradation of phenol by anaerobic MICP, with strain CZ3 showing significant removal of nitrate, fluoride, calcium, and phenol. Several scholars [22] have investigated the effects of different carbon to nitrogen ratios on the biological removal of aquatic pollutants such as calcium (Ca^2+^), fluorine (F^−^), and nitrate (NO_3_-N) in quartz sand-filled biofilm reactors (QSBRs).

The factors affecting the micro-morphology and crystallinity of mineralisation products during the MICP process still need to be studied in depth. In this paper, the effect of different bacteria-to-calcium ratios on the biomineralisation product CaCO_3_ was investigated for the first time using two methods of introducing calcium sources in the MICP process: calcium-first and calcium-later addition. Ca^2+^ in calcium acetate was biomineralised by the MICP technique. First, a culture of *Sporosarcina pasteurii* was carried out to study the activity of the bacterial solution used for MICP in this experiment. Then, the sequence of calcium source introduction with different bacteria-to-calcium ratios was designed, and its effect on the yields of the mineralised products was investigated. To further explore the various properties of the mineralised products, XRD, SEM, nanoindentation, and TG-DSC were used. Subsequently, it was proposed that the crystallinity and micromorphology of CaCO_3_ could be regulated by changing the order of introduced calcium sources and adjusting the bacteria-to-calcium ratio.

## 2. Materials and Methods

### 2.1. Experimental Materials

The original strain was *Sporosarcina pasteurii* DSM 33 from the German Conservation Centre for Microorganisms and Cell Cultures (German Conservation Centre for Microorganisms and Cell Cultures, Bonn, Germany), and the medium composition was 15.0 g of casein peptone (Sinopharm Chemical Reagent Co., Shanghai, China), 5.0 g of soy peptone (Sinopharm Chemical Reagent Co., Shanghai, China), 5.0 g of NaCl (Beichen Fangzheng Reagent Factory, Tianjin, China), 20.0 g of urea (Beichen Fangzheng Reagent Factory, Tianjin, China), and 1000.0 mL of deionised water, with an additional 15.0 g of agar (Sinopharm Chemical Reagent Co., Shanghai, China) to be added to the solid medium. Urea was analytically pure with a molecular weight of 60.06, and the additional calcium source (Beichen Fangzheng Reagent Factory, Tianjin, China) was analytically pure calcium acetate with the molecular formula C_4_H_6_CaO_4_·H_2_O and a molecular weight of 176.18.

### 2.2. Bacterial Growth

The bacterial culture process was carried out in a sterile room. A solid medium and liquid medium were prepared, and the pH of the medium was adjusted to 7.3 using 1 mol/L NaOH solution (Beichen Fangzheng Reagent Factory, Tianjin, China), which was put into Petri dishes and triangular flasks, respectively, and sterilised at 0.13 MPa, 121 °C, and 25 min for use. The ampoule bottle powder of bacteria was removed from the ampoule tube, inoculated onto the solid medium, and activated at a temperature of 30 °C for 48 h. The activated bacteria were picked and inoculated into the liquid medium of the triangular flasks (pH = 7.3), which was recorded as the first-generation bacterial solution. The first generation of bacterial liquid was divided into test tubes and incubated aerobically in a constant temperature water bath shaker with the parameters of 30 °C and 170 r/min for 24 h, and then the first generation of bacterial liquid was taken in equal quantities with a pipette gun and inoculated into triangular flasks according to 12% of the inoculum volume for the second generation of bacterial liquid culture for 24 h. The second generation of bacterial liquid was used in the present study for the experiments.

### 2.3. Sequence of Introduction of Calcium Sources and Design of Bacteria-to-Calcium Ratio

**Figure 1:**
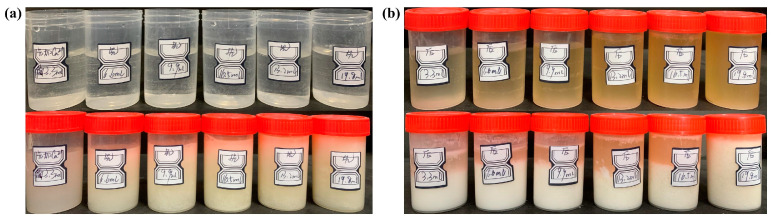
Mineralization of different bacteria-to-calcium ratios under different calcium addition sequences. (**a**) Reaction phenomena under the condition of adding calcium first in Group A. (**b**) Reaction phenomena under the condition of adding calcium later in Group B.

Assuming that urea can be completely decomposed, 30 mL of a 1 mol/L urea solution contains 1.8018 g of urea, and complete decomposition will yield 3 × 10^−2^ mol ${\mathrm{CO}}_{3}^{2-}$; thus, 3 × 10^−2^ mol Ca^2+^ or a 5.285 g calcium acetate test sample is required. Twelve test samples with this mass of calcium acetate were weighed and set aside. A 1 mol/L urea solution was prepared in 12 reaction bottles, each set to 30 mL, divided into two groups, A and B. Group A was set as the calcium addition first group by mixing the urea solution with calcium acetate thoroughly, then adding the bacterial solution, and then filtering, drying, and weighing after 48 h of resting. Group B was the later calcium addition group; the urea solution was mixed with the bacterial solution, followed by 24 h of rest, and then the calcium acetate was added to the solution. The solution was allowed to rest for 24 h and was then filtered, dried, and weighed. There were six bacteria-to-calcium ratios: 1/9, 2/9, 3/9, 4/9, 5/9, and 6/9, and the amount of bacterial solution added was 3.3 mL, 6.6 mL, 9.9 mL, 13.2 mL, 16.5 mL, and 19.8 mL, respectively. When the conductivity was measured in this study, the ratio of the bacterial solution to the 1 mol/L urea solution was 1/9, and for the purpose of the control test, the starting bacteria-to-calcium ratio was set to 1/9. Calcium-first and calcium-later addition modes are shown in Figure 1.

### 2.4. Measurement Methods

The microbial activity was characterised by the rate of change in the conductivity of the bacterial solution Δ*κ/*Δ*t* and the value of OD_600_ together. The rate of change in conductivity was measured by taking 18 mL of 1 mol/L urea solution and mixing it with 2 mL of the bacterial solution, measuring the difference in conductivity of the mixed solution for 5 min by using a multiparameter metre (DZS-708 L), and then calculating the rate of change in bacterial solution conductivity in μs/cm/min. The OD_600_ value of the bacterial solution was measured using ultraviolet spectrophotometry (UV-1800) when the first measurement was greater than 0.8 Abs, and a second measurement was carried out for the sake of the accuracy of the measurement value. The measurement was carried out by diluting the bacterial solution by a certain number of times using deionised water, and when the measurement value was less than 0.8 Abs, the measurement value and the number of dilutions were multiplied to obtain the OD_600_ value of the bacterial solution. The calcium carbonate yield *η* was analysed using the ratio of the actual production *m_s_* to the theoretical production *m*_0_. The morphology and chemical elemental composition of the products were analysed by SEM (TESCAN MIRA4) and EDS (Oxford Xplore30). The crystal structures of the products were analysed using XRD (Empyrean Sharp X-ray Diffraction System, Malvern Panaco, Almelo, The Netherlands). The products were analysed by TG-DSC (NETZSCH Synchro Thermal Analyser STA 449 F5, Selb, Germany) at a temperature increase rate of 10 K/min. The temperature was set from 35 to 1000 °C using an Al_2_O_3_ crucible and N_2_ atmosphere. Nanoindentation (Nano Test Vantage, a comprehensive mechanical properties testing system for MML (micro) nanomaterials, Wrexham, UK) was used to characterise the mechanical properties of the products.

## 3. Results and Discussion

### 3.1. Effect of Calcium Addition Method and Bacteria-to-Calcium Ratio on Sediment CaCO_3_ Yield

After measuring the microbial activity, the bacterial solution Δ*κ/t* was 50 μs/cm/min, and the OD_600_ value was 1.0 Abs. By observing the reaction phenomenon (Figure 1), it was found that the deposition rate of the mineralisation products in the reaction bottles of Group A was slower, and the products were flocculent and suspended in the solution, with a yellowish colour, and were slowly deposited at the bottom of the bottles. The delamination of the solution with the mineralisation products was not obvious, and the deposition rate of the product in the bottle of Group B was significantly greater than that of Group A. The mineralised product was white and rapidly deposited at the bottom of the reaction bottle, and the stratification between the solution and the mineralised product was obvious. The above phenomenon is due to the introduction of the calcium source in different orders. In the Group A mixed solution, bacterial decomposition of urea and sediment generation occurred simultaneously, and this process can be interpreted as Ca^2+^ waiting for ${\mathrm{CO}}_{3}^{2-}$. In Group B, bacterial decomposition of urea took place in the first 24 h. After 24 h, the solution already contained a large amount of ${\mathrm{CO}}_{3}^{2-}$. After adding calcium acetate, dissolved Ca^2+^ and ${\mathrm{CO}}_{3}^{2-}$ quickly combined to produce a precipitate. Similarly, this process can be interpreted as ${\mathrm{CO}}_{3}^{2-}$ waiting for Ca^2+^.

**Figure 2:**
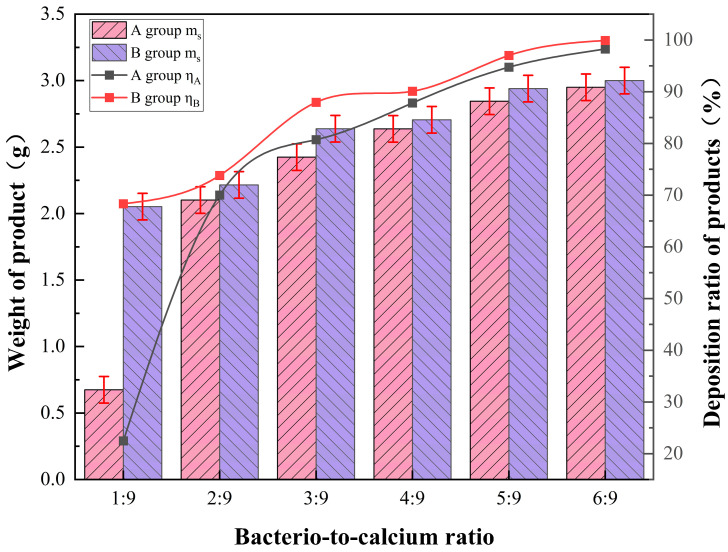
Effect of bacteria-to-calcium. ratio on the yield and ratio of production of mineralised products under different calcium addition sequences.

According to Figure 2, it can be seen that the mineralisation product yield *η* was only 22.48% when the bacteria-to-calcium ratio of Group A was 1:9, and when the bacteria-to-calcium ratio was increased to 2:9, the mineralisation product *η* was elevated by approximately 2.11 times, and *η* reached 70.01%. At the same change in the bacteria-to-calcium ratio, Group B did not show a large difference in yield. Under the condition of a certain ratio of urea and calcium source, with the increase in the bacteria-to-calcium ratio and the addition of bacterial liquid, the yield of mineralised product *m_s_* of both Group A and Group B increased, and the yield *η* increased; when the bacteria-to-calcium ratio reached 6:9, the *η* of both Group A and Group B was close to 100%. This phenomenon can be attributed to the calcium-first mode. When the bacteria-to-calcium ratio is small, the ${\mathrm{CO}}_{3}^{2-}$ from urea decomposition gradually combines with Ca^2+^ to form CaCO_3_ encapsulated on the surface of the bacteria, which hinders the bacteria from further producing urease and prevents the further decomposition of urea. But with the increase in the bacteria-to-calcium ratio, this phenomenon is no longer an obstacle to the decomposition of urea, and the yield and rate of CaCO_3_ production are close to those of the later calcium addition mode.

### 3.2. Crystal Structure, Morphology and Elemental Analysis of Mineralisation Products

**Figure 3:**
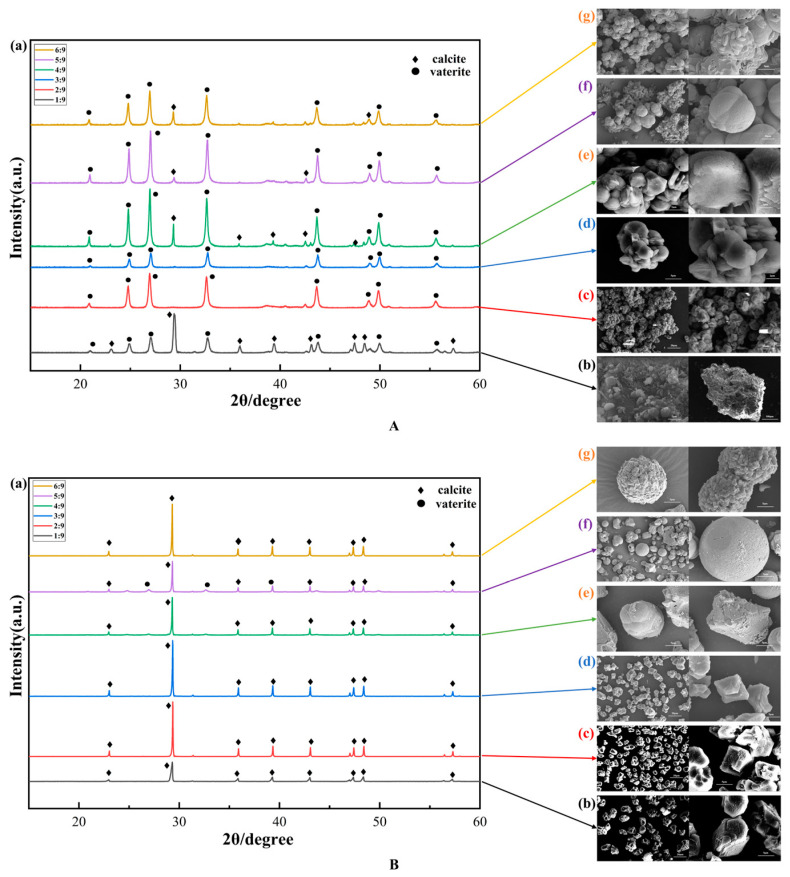
X-ray diffraction patterns and SEM images of mineralisation products. (**A**) is the calcium-first addition method, and (**B**) is the calcium-last addition method. (**a**) X-ray diffraction patterns at different bacteria-to-calcium ratios, and (**b**–**g**) represent SEM images with bacteria-to-calcium ratios of 1/9~6/9, respectively.

According to Figure 3A(a), two distinct calcium carbonate polymorphs, calcite and vaterite, appeared in the mineralisation products for different bacteria-to-calcium ratios under the calcium-first addition mode. At the bacteria-to-calcium ratio of 1/9, the mineralisation products are dominated by calcite as the main crystalline phase, with the presence of a small amount of vaterite, with a strong diffraction peak at 2θ = 29.4°, corresponding to the diffraction plane (104), and cell parameters of a = 4.990, b = 4.990, and c = 17.061. At bacteria-to-calcium ratios of 2/9 and 3/9, the main crystalline phase of both mineralisation products is vaterite, with strong diffraction peaks occurring near 2θ = 26.9° and 32.6°. The mineralisation products at bacteria-to-calcium ratios of 4/9, 5/9, and 6/9 all have vaterite as the main crystalline phase, with a small amount of calcite formation. Under calcium-first addition, with the increase in the bacteria-to-calcium ratio, the mineralisation products changed from the calcite to the vaterite, and the intensity of vaterite diffraction peaks was generally higher than that of calcite diffraction peaks, which indicated that the amount of bacterial liquid added could be used to regulate the crystalline shape of microbial mineralisation deposition products under the calcium addition first mode. As seen from Figure 3A(b), when the bacteria-to-calcium ratio is 1/9, the microbial mineralisation product particles accumulate in a pile with sizes ranging from 300 to 400 μm and are more densely packed. The surface of the piled-up particles is attached by smaller-sized calcium carbonate, which exists in a variety of morphologies, such as irregular, spherical, flattened, and polyhedral. According to Figure 3A(c,d), when the bacteria-to-calcium ratio is 2/9 and 3/9, the stacking size of the mineralisation product particles is between 10 and 30 μm, the calcium carbonate has a more flat and flaky structure, the stratification between particles is obvious, and the particles show irregular stacking status. According to Figure 3A(e), when the bacteria-to-calcium ratio is 4/9, the calcium carbonate particles are clearly separated from each other, the shape is more irregular, the particles with different shapes are embedded in each other, the stacked particles are smaller than 10 μm, and the surface of the particles is rough and there are holes, which may be caused by the death of bacteria during the process of microbial mineralisation. As shown in Figure 3A(f), when the bacteria-to-calcium ratio is 5/9, the mineralisation products either accumulate in lamellar or laminar structures or in individual particles in the form of pumpkin-shaped or spherical accumulations. The surface of pumpkin-shaped particles had light lines without small particles attached. The surface of spherical particles was rough with laminar structure and small particles of mineralisation products attached, and the spherical particles were stacked more loosely than the laminar particles. As shown in Figure 3A(g), when the bacteria-to-calcium ratio is 6/9, the mineralisation products mostly appear as spherical or ellipsoidal particles accompanied by a small number of lamellar particles. The size of individual spherical particles is larger than 10 μm, and uniformly distributed scale-like products appear on the surface.

According to Figure 3B(a) in Figure 3, it can be seen that under the condition of later calcium addition, the main crystal type of the mineralisation products of the six groups of tests is calcite. The characteristic main peak appeared at 29.4°, and the positional peak intensity was very obvious, indicating that in the crystallisation process of calcium carbonate (104), the mirror energy was the lowest, which was suitable for the synthesis of crystals; except for a small amount of vaterite-type calcium carbonate generated when the bacteria-to-calcium ratio was 5/9, the rest of the groups were all calcite-type calcium carbonate, and the purity was higher, which indicated that the bacterial liquid addition had less effect on the crystalline phase of the mineralisation products under the condition of later calcium addition, and therefore, the later calcium addition indicates that the addition of calcium to the bacterial solution under later calcium addition conditions has less effect on the crystalline phase of the mineralised products, and therefore, the later calcium addition method appears to stabilise the synthesis of calcite calcium carbonate. From Figure 3B(b–d), it can be seen that the mineralisation products are all irregular polyhedral blocks with rough surfaces when the bacteria-to-calcium ratios are 1/9, 2/9, and 3/9, and the particle sizes are basically in the range of 5–10 μm, and they are uniformly distributed, showing no obvious stacking. Individual particles are blocky as a whole, with a laminated structure and obvious interlayer texture. As seen from Figure 3B(e), when the ratio of bacteria/calcium is 4/9, the mineralisation products are composed of spherical and irregular polyhedral shapes, with particle sizes between 5 and 20 μm; the number of spherical particles and massive particles is similar; micropores appear on the surface of some of the spherical particles; and there is accumulation of the particles. Spherical calcium carbonate is divided into the “pumpkin type” with a smooth surface and the surface microporous calcium carbonate. There are two kinds of spherical calcium carbonate: the “pumpkin type”, with a smooth surface, and calcium carbonate, with a microporous surface. The layered structure of lumpy particles is obvious, and a small amount of particles with different shapes are cemented together. As seen from Figure 3B(f), under the condition of a 5/9 ratio of bacteria/calcium, the mineralisation product is spherical or has polyhedral lumps, accompanied by the appearance of spherical microporous calcium carbonate. The spherical calcium carbonate particle sizes ranged from 5 to 20 μm, with a uniform distribution of nanoscale micropores on the surface and the individual occurrence of larger micron-sized pores, which reflect the influence of bacteria on the morphology of calcium carbonate during its growth. The blocky particles were shown to consist of lamellar or laminar stacks. From Figure 3B(g), it can be seen that the morphology of the mineralisation product is spherical and irregular polyhedral lumps when the bacteria-to-calcium ratio is 6/9. The lumpy product is similar to the previous one, and the lumpy calcium carbonate is built up from the lamellar structure and piled up to form the mineralisation product with a larger particle size. The spherical calcium carbonate morphology changes dramatically; the surface of spherical calcium carbonate can be observed to consist of a large number of irregular blocks, and adjacent particles are embedded in each other, which shows that blocky calcium carbonate with small particle sizes can be assembled and stacked to form large particle products with different shapes.

In summary, under the condition of adding calcium first in Group A, with the increase in the bacteria-to-calcium ratio, the deposition rate of mineralisation products accelerated, the morphology of microbial mineralisation products decreased in terms of particle diameter size, the morphology changed, the degree of accumulation gradually weakened, and the mineralisation products gradually changed from a small pieces of a whole to a powder. This indicates that the deposition rate of mineralisation products is related to the decomposition rate of urea, and the mineralisation deposition rate is different, resulting in changes in the morphology of mineralisation products. In the case of Group B with later calcium addition, the degree of accumulation of mineralisation products was generally weak, mostly in the form of dispersed particles. With the increase in the bacteria-to-calcium ratio, the mineralised products showed a special morphology, i.e., spherical calcium carbonate with micropores and spherical calcium carbonate made of microblocks. Under the same bacteria-to-calcium ratio, the order of introduction of the calcium source has a greater influence on the shape of mineralised products, and the degree of accumulation in Group A is generally higher than that in Group B. Vaterite is predominant in Group A, while irregular lumpy calcite is predominant in Group B. The samples in Groups A and B were examined for their appearance. EDS spectroscopic analysis of the samples in Groups A and B showed that the elements in the mineralisation products are C, O, and Ca.

### 3.3. Mechanical Properties of Nanoindentation of Mineralisation Products

**Figure 4:**
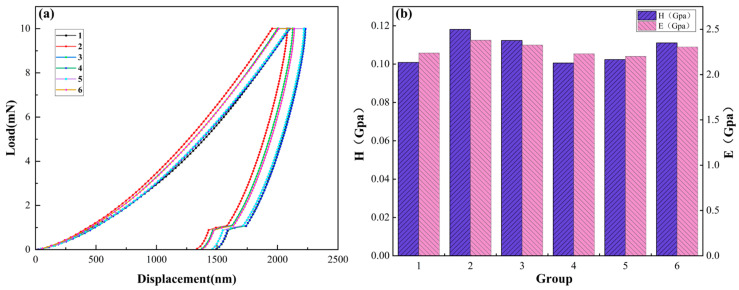
Nanoindentation test results of mineralised products at different bacteria-to-calcium ratios. (**a**) Mineralisation product load-down indentation depth curves. (**b**) Calculated results of mineralisation product hardness H and elastic modulus E. 1~6 represent bacteria-to-calcium ratios of 1/9~6/9, respectively.

In nanoindentation experiments, after the indenter is gradually pressed into the sample, the material near the indenter first deforms elastically, and as the load increases, the sample begins to deform plastically, and an indentation matching the shape of the indenter appears in the sample. When the indenter is unloaded, the elastic deformation is recovered, and the plastic deformation forms an indentation crack. The nanoindentation load-depth variation curve was plotted from the experimental data, as shown in Figure 4a, and the curve was used to calculate the elastic modulus (*E*) and hardness (*H*) of the mineralised product, as shown in Figure 4b.

Using the tested load-down depth curve, the hardness and elastic modulus of the material can be obtained by the Oliver–Pharr (O&P) method [23,24]. In the O&P method, the contact depth *h_c_* is calculated by the unloading curve using Equation (7):

(7) $$h_{c}=h-\varepsilon \frac{P(h)}{S}$$

where *h_c_* is the maximum indentation depth in nm, *P* is the corresponding load in mN, *ε* is a modification constant (*ε* = 0.75 for Berkovich indenter), and *S* is the slope of the unloading curve (*S* = d*P*/d*t*). Then, according to the contact area function Equation (8):

(8) $$A_{c}=24.56h_{c}^{2}+\alpha_{1}h_{c}+\alpha_{2}h_{c}^{1/2}+\cdots +\alpha_{8}h_{c}^{1/128}$$

For a Berkovich indenter, *A_c_* = 24.5, the hardness (*H*) and the approximate modulus of elasticity (*E_r_*) of the material can be calculated. The calculation formulae are as follows (9) and (10):

(9) $$H=\frac{P}{A_{c}}$$

(10) $$E_{r}=\frac{S\sqrt{\pi }}{2\sqrt{A_{c}}}$$

The load *P* in Equation (3) is taken at the maximum displacement. The relationship between the approximate modulus of elasticity *Er* and the modulus of elasticity *E* of the material is given in Equation (11):

(11) $$\frac{1}{E_{r}}=\frac{1-v^{2}}{E}+\frac{1-v_{i}^{2}}{E_{i}}$$

where *v* and *v_i_* are the Poisson’s ratio of the material and indenter, respectively; *E* and *E_i_* are the modulus of elasticity of the material and indenter, respectively, and *E_i_* = 1141 GPa, *v_i_* = 0.07, and *v* = 0.28 were taken during the calculation.

In this paper, the research aim is mainly to investigate the modulation of the CaCO_3_ crystalline form and mechanical properties at micro and nano scales only for the mineralisation products with different bacteria-to-calcium ratios under the Group B calcium addition method. The indentation depth of the mineralisation products was 1200 nm~1500 nm, as shown in Figure 4a, which did not show that the change in indentation depth was affected by the change in the bacteria-to-calcium ratio. In terms of the mechanical properties of materials at the micro- and nanoscale, for nanoindentation technology, the indentation position, maximum load, contact stiffness, etc., will affect the test results; it is difficult to ensure the mechanical properties of the same material under different test conditions in the experimental process, but nanoindentation technology is still a method for determining the mechanical properties of materials at the micro- and nanoscale. After calculation, it can be seen that the average nanoindentation hardness and elastic modulus of CaCO_3_, the mineralisation product of the later calcium addition method, were *H* = 0.11 GPa and *E* = 2.28 GPa, respectively. This suggests that the mechanical properties of microbial mineralisation products on the micro- and nanoscale are less affected by the bacteria-to-calcium ratio.

### 3.4. Mineralisation Product TG-DSC Analysis

**Figure 5:**
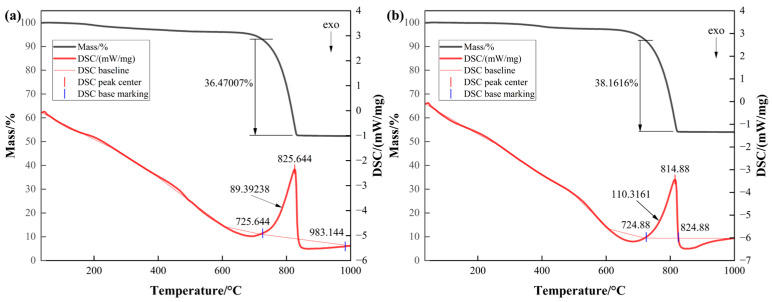
Mineralisation product TG-DSC test results. (**a**) TG-DSC curves of mineralisation products under the condition of adding calcium first in Group A. (**b**) TG-DSC curves of mineralisation products after adding calcium later in Group B.

As seen from Figure 5a, under a N_2_ atmosphere, the calcium carbonate produced by the calcium first addition method has a small decrease in the curve before the starting temperature to 725.644 °C, which is due to the mass loss of the bound water in the product, which starts to have a mass loss when the temperature is heated up to 725.644 °C. The decomposition ends at a temperature of 825.644 °C, and there is a clear heat absorption peak, with the amount of heat absorbed being 89.39 J/g, and the mass loss rate was approximately 36.47%. From Figure 5b, it can be seen that the calcium carbonate produced by the later calcium addition method began to decompose when the temperature was heated to 724.88 °C, and the decomposition ended when the temperature was 814.88 °C.

There was an obvious heat absorption peak, with a heat absorption of approximately 110.32 J/g, and the mass loss was approximately 38.16%. Comparing the thermal decomposition temperatures of the mineralisation products generated by A and B, it was found that the decomposition temperatures of the two were close to each other, and there was little difference in the thermodynamic parameters such as mass loss. This shows that the thermodynamic parameters of the microbial mineralisation product CaCO_3_ are less affected by the order of calcium addition and the change in the bacteria-to-calcium ratio. Combined with the previous research in this paper, it was found that the introduction order of the calcium source and bacteria-to-calcium ratio have a greater impact on the yield of the mineralisation product and can regulate the crystalline shape and microscopic morphology of the product, but the micromechanical properties and thermodynamic parameters of the different crystalline and microscopic morphologies of CaCO_3_ do not differ much.

## 4. Conclusions

By changing the order of introducing the calcium source and controlling the ratio of bacteria to calcium, the MICP process can be realised to regulate the crystalline shape and microscopic morphology of CaCO_3_, but it does not have much effect on its micromechanical properties and thermodynamic parameters. When MICP was carried out by adding calcium first and the ratio of bacteria to calcium was 2/9 and 3/9, a large amount of vaterite-type CaCO_3_ could be produced stably, which can be used for research on drug carriers, etc. When calcium was added later, a large amount of calcite-type CaCO_3_ could be produced with different ratios of calcium to bacteria, which can be used for research in areas such as repairing cracks in concrete. Future research on the application of MICP to repair concrete cracks can be carried out by investigating its repair method, repair effect, and bonding mechanism between mineralisation products and concrete.

In conclusion, this study can provide some theoretical basis for regulating calcium carbonate, the mineralisation product of MICP, and some guidance for the application of MICP in practical engineering.

## Data Availability

The data are not publicly available due to privacy.
